# Targeting synthesis of the Chromosome Replication Initiator Protein DnaA by antisense PNA-peptide conjugates in *Escherichia coli*

**DOI:** 10.3389/frabi.2024.1384390

**Published:** 2024-04-08

**Authors:** Christopher Campion, Godefroid Charbon, Peter E. Nielsen, Anders Løbner-Olesen

**Affiliations:** ^1^ Department of Cellular and Molecular Medicine, University of Copenhagen, Copenhagen, Denmark; ^2^ Department of Biology, University of Copenhagen, Copenhagen, Denmark

**Keywords:** novel antibiotics, antisense PNA-peptide, chromosome replication, DnaA initiator protein, inhibition of initiation, *Escherichia coli*

## Abstract

Initiation of chromosome replication is an essential stage of the bacterial cell cycle that is controlled by the DnaA protein. With the aim of developing novel antimicrobials, we have targeted the initiation of DNA replication, using antisense peptide nucleic acids (PNAs), directed against DnaA translation. A series of anti-DnaA PNA conjugated to lysine-rich bacterial penetrating peptides (PNA-BPPs) were designed to block DnaA translation. These anti-DnaA PNA-BPPs inhibited growth of wild-type *Escherichia coli* cells at low micromolar concentrations, and cells exposed to anti-DnaA PNA-BPPs exhibited characteristic hallmarks of chromosome replication inhibition. These results present one of very few compounds successfully targeting initiation of chromosome replication, an essential step in the bacterial cell cycle.

## Introduction

The burden of increased antibiotic resistance has led to a growing, urgent demand for the discovery and development of novel antibiotic therapeutic strategies. Peptide nucleic acid (PNA) is a potent DNA mimic, with the potential to provide an avenue to antisense drugs combatting bacterial pathogens (Good et al., 2001; Ghosal, 2017; Lee et al., 2019; Saarbach et al., 2019; Wojciechowska et al., 2020; Iubatti et al., 2022). In PNA, the sugar backbone of natural nucleotides is replaced by a neutral pseudo-peptide backbone, consisting of 2-amino-ethyl-glycin (aeg) units (Nielsen et al., 1991). PNA forms stable duplexes with sequence complementary DNA and RNA, based on the Watson–Crick hydrogen recognition (Egholm et al., 1993), with higher stability than duplexes formed by natural phosphodiester backbone nucleic acids. For antimicrobial approaches, PNA is usually designed as 10–12-nucleobases long PNA oligomers to target mRNA transcripts of essential genes. A region between the start codon and the ribosomal binding site is most frequently used as a PNA target site, as this leads to steric hindrance of translation initiation (Good et al., 2001; Dryselius et al., 2003; Goltermann et al., 2019). Unmodified PNA is not readily taken up by bacterial cells (Good and Nielsen, 1998; Good et al., 2000) but must be conjugated to a delivery moiety such as a bacterial-penetrating peptide (BPP) (Good et al., 2001) to translocate across the bacterial envelope. Subsequently, antimicrobial PNA-BPPs against many pathogens using a variety of BPP conjugates and targeting a variety of genes have been described (Ghosal, 2017; Narenji et al., 2017; Lee et al., 2019; Campion et al., 2021).

Replication of the *E. coli* chromosome is initiated from *oriC*, from which the two nascent replication forks proceed bi-directionally, reaching completion at the terminus, *ter* region, after which two fully replicated chromosomes are segregated to daughter cells (for reviews, see Wolanski et al., 2014; Frimodt-Møller et al., 2024). The highly conserved DnaA protein initiates DNA replication by binding to specific “DnaA-boxes” in *oriC*, thereby facilitating strand opening at an AT-rich “DNA unwinding element, DUE” region (Bramhill and Kornberg, 1988), where two replisomes are subsequently assembled (Frimodt-Møller et al., 2024). The *E. coli* DnaA protein contains four domains: an N-terminal protein interaction domain (Domain I); a linker domain (Domain II); an AAA+ ATPase domain, responsible for ATP/ADP binding and ATP hydrolysis (Domain III); and a double-stranded DNA binding domain (Domain IV) (Hansen and Atlung, 2018; Frimodt-Møller et al., 2024).

Only the ATP-bound form of DnaA (DnaA^ATP^) is active in initiation (Sekimizu et al., 1987) and binds to low-affinity DNA-boxes in *oriC*, forming a structure causing a superhelical tension at the DUE region, which in turn results in DNA strand opening, that is, open complex formation (Erzberger et al., 2006; Ozaki et al., 2008). DnaA subsequently recruits two hexameric DnaB helicase complexes to the DUE region, which eventually leads to the assembly of two replisomes (Sakiyama et al., 2022). (For a thorough review of oriC structure and interactions with DnaA and other initiation factors, see Frimodt-Møller et al., 2024). It is essential that the initiation of chromosome replication is tightly controlled in the cell cycle, as the inability to initiate chromosome replication leads to chromosome loss (Botello and Nordstrom, 1998), whereas uncontrolled initiations are detrimental to genome integrity and result in chromosome breaks and cell death (Simmons et al., 2004; Charbon et al., 2014). It is therefore not surprising that the initiation frequency is controlled by a stringent system of mechanisms to ensure that initiation occurs only once per cell cycle, and simultaneously from all cellular origins (Skarstad et al., 1986). For thorough reviews on mechanisms controlling the initiation of DNA replication in *E. coli*, see Katayama et al. (2017), Hansen and Atlung (2018), and Frimodt-Møller et al. (2024).

In the present study, we designed and characterized antisense PNA-BPPs targeting translation of the chromosomal replication initiator protein, DnaA. We show that *E. coli* is sensitive to DnaA-targeting PNA-BPPs in the micromolar range. PNA-BPP target specificity was verified directly by Western blotting and indirectly by a DnaA-inhibitor assay. Cell cycle parameters obtained by flow cytometry and *in vivo* fluorescent microscopy all revealed phenotypic hallmarks of a true inhibitor of chromosomal replication initiation.

## Materials and methods

### Bacterial growth conditions

Cells were grown at 37°C in non-cation adjusted Mueller–Hinton broth (MHB-I; Sigma Aldrich, Darmstadt, Germany), Lysogeny Broth (LB), or in AB minimal medium (Clark and Maaløe, 1967) supplemented with 10 μg/mL of thiamine, 0.2% glucose or 0.2% glycerol and 0.5% casamino acids. Antibiotics were used at the following concentrations: ampicillin (150 μg/mL), chloramphenicol (20 μg/mL), kanamycin (50 μg/mL), streptomycin (100 μg/mL), tetracycline (10 μg/mL), cephalexin (36 μg/mL), and rifampicin (300 μg/mL).

### Bacterial strains

All strains used in this study are listed in **Supplementary Table 1**. ALO8290 was created by the P1 transduction of *dnaA46* with *tnaA:Tn10* into ALO4223 using a P1 lysate of the strain ALO2342 and selecting for tetracycline resistance and screened for thermo-sensitivity.

The R1-based plasmid pJEL-*dnaA*, was constructed by amplifying the *dnaA* gene, including promoter regions (*dnaA1p* and *dnaA2p*) of MG1655 using primers 5′-CCAGAAGCTTAAGCCAATTTTTGTCTATGG-3′ and 5′-CCAGGGATCCGTTGTAGCGGTTTTAATAAA-3′. The PCR product was digested with *Hin*dIII and *Bam*HI and ligated into plasmid pJEL109 (Lobner-Olesen et al., 1992), previously digested with the same enzymes. ALO8528, used for DnaA depletion, was created by deletion of *dnaA* in MG1655 carrying plasmids pJEL-dnaA and pKG339 (Jensen et al., 1995) by P1 transduction, using a lysate of the strain TC3478 carrying the *ΔdnaA::cat* mutation (Ingmer and Atlung, 1992).

### Antisense PNA design, synthesis, and handling

Antisense PNA were designed as 10-mer nucleobase oligomers, to complement the region between the Shine-Dalgarno sequence and the start codon on the *dnaA* mRNA transcript of the strain MG1655 (NCBI: ref. NC_000913.3) (**Table 1**). Selected sequences were checked for target specificity to *dnaA* using the NCBI BLASTn software (https://blast.ncbi.nlm.nih.gov/Blast.cgi; **Supplementary Table 2**). PNA-BPPs were synthesized by the continuous standard peptide solid-phase peptide synthesis (SPPS), with tBoc protected monomers, on an MBHA resin. The lysine-rich (KFF)_3_K carrier peptide was added in synthesis to the N-terminal of the PNA, via an eg1 (8-amino-3,6-dioxaoctanoic acid) linker. PNA-BPP conjugates were purified and verified by HPLC and MALDI-TOF mass spectrometric analysis (**Supplementary Figure S1**), as described previously (Christensen et al., 1995). All PNA-BPP handling was performed as described in detail (Goltermann and Nielsen, 2020).

**Table 1:** PNA-BPP conjugates.

PNA	Sequence including BPP (N-C)	Location to target gene (5′-3′)	MIC (μM)
DnaA-1-PNA	H-(KFF)_3_K-eg1-TCCACTCGAA-NH_2_	UUCGAGUGGAGUCCGCC**GUG**	4
DnaA-2-PNA	H-(KFF)_3_K-eg1-CTCCACTCGA-NH_2_	UUCGAGUGGAGUCCGCC**GUG**	4
DnaA-3-PNA	H-(KFF)_3_K-eg1-ACTCCACTCG-NH_2_	UUCGAGUGGAGUCCGCC**GUG**	2
Mismatch PNA	H-(KFF)_3_K-eg1-ACCCCATTCG-NH_2_	Na	16

Complete sequences of anti-DnaA PNA-BPPs along with their binding target on the mRNA. The dnaA start codon is highlighted with bold; the PNA binding position is underlined. PNA-BPPs were synthetized as described (Yavari et al., 2021).

Na, not applicable; eg1, 8-amino-3,6-dioxaoctanoic acid.

### Minimal inhibitory concentration

*E.coli* susceptibility to anti-DnaA PNA-BPPs was determined on cultures diluted to OD = 0.0002 by broth microdilution, using a Clinical and Laboratory Standards Institute (CLSI) protocol updated for PNA use, as described previously (Goltermann and Nielsen, 2020). Since PNA-BPPs adhere to polystyrene surfaces, this updated protocol includes the use of low-binding plastic, non-cation-adjusted Mueller–Hinton broth, alongside PNA-BPP stocks and dilution prepared in 0.4% BSA/0.02% acetic acid. MICs were determined with a BIOTEK™ Synergy H1 microplate reader (Agilent, Santa Clara, CA, USA).

### Western blot analysis

Wild-type cells were grown exponentially in AB minimal medium supplemented with glucose and casamino acids, diluted to OD_450 _= 0.01 and exposed to PNA-BPPs. Cells were collected and harvested after 3 h. Harvested cells were washed in 10-mM Tris-HCl pH 8 and 10-mM MgCl_2_ and kept at −20°C. Samples were heated at 95°C for 5 min and sonicated using a Branson 450 sonifier. Total protein content was quantified using Bradford Reagent (Sigma Aldrich), normalized to the same protein level (1–1.5 μg) and separated using SDS-PAGE (Precast Gel: 10–20% Tris-HCL: Bio-Rad Inc., Copenhagen, Denmark) in an Xcell 4 SureLock™, Midi-Cell (ThermoFisher Scientific, Waltham, MA, USA). Samples were transferred to a polyvinylidene difluoride (PVDF) membrane (Whatman™, GE Healthcare, Chicago, IL, USA), using a semidry blotting apparatus (JKA Biotech, Denmark). DnaA protein was detected with polyclonal anti-DnaA antibodies (Charbon et al., 2021). The PVDF membrane was incubated with Enhanced Chemiluminiscense (ECL) substrate (Bio-Rad) and the signal was detected using an ImageQuant LAS400 (GE Healthcare, Life Sciences). Quantification of Western blotting and analysis was performed using ImageJ software. A nonspecific band seen with the DnaA antibody was used as loading control and is included in the figures.

### Inhibitor assay

The reporter strain (ALO5429) was grown overnight in AB minimal medium supplemented with glucose and casamino acids, and with appropriate antibiotics (40 μg/mL of kanamycin, 20 μg/mL of chloramphenicol, and 50 μg/mL of streptomycin). The strain was diluted to 1–5 × 10^5^ CFU/mL and added as 90-μl aliquots to a clear-bottomed black-sided 96-well plate already containing 10 μL of DnaA-3-PNA at the desired range of concentrations. The plate was incubated for 18 h at 37°C, while continuously shaken at 220 rpm, in a BIOTEK™ Synergy H1 microplate reader. After 18 h, OD_450_ and fluorescence (485-nm excitation and 528-nm emission) were measured in the BIOTEK™ Synergy H1 microplate reader. The fluorescence of each well was compared to the fluorescence of the untreated wells. A control strain (ALO5125) without the lambda *cI* gene carrying mini-chromosome pRNK6 was included in the experiments as reference for maximal green fluorescent protein (GFP) fluorescence.

### Flow cytometry

Cell cycle parameters were determined by flow cytometry using an Apogee A10 instrument, as previously described (Lobner-Olesen et al., 1989). Samples for flow cytometry were taken from cultures prior to and following treatment with rifampicin/cephalexin. Briefly described, 1 mL of cell samples was centrifuged and fixed in 70% ethanol and 100-μL 10-mM Tris buffer at pH 7.5 and then stored at 4°C. The number of origins per cell was determined from a sampled treated with rifampicin (300 μg/µL) and cephalexin (36 μg/µL) for 4 h prior to fixation. Rifampicin inhibits DNA replication indirectly through the inhibition of RNA synthesis; however, ongoing replication cycles will be completed, while cephalexin prevents cell division. Therefore, the number of chromosomes per cell will represent the number of origins of replication present at the time of rifampicin/cephalexin addition.

### *In vivo* visualization of the origin and terminus by fluorescence microscopy

Cells were washed and resuspended in 0.9% NaCl and kept on ice until mounted on microscope pads coated with a 1% agarose. Fluorescence microscopy was performed using an AxioImager Z1 microscope (Carl Zeiss MicroImaging, Inc., Jena, Germany), with a 100× objective and a Hamamatsu ORCA-ER C4742-80-12AG camera, as described previously (Charbon et al., 2014). Images were processed with ImageJ Software (Schneider et al., 2012; Schindelin et al., 2015).

### DnaA depletion

Strain ALO8528 was grown exponentially in AB minimal medium supplemented with glycerol, casamino acids, and 10 μg/mL of tetracycline. At time T = 0, 1 mM of isopropyl beta-D-1 thiogalactopyranoside (IPTG) was added to the growth medium to initiate DnaA depletion. During depletion, the cultures were diluted in fresh pre-warmed medium containing IPTG.

### Statistical analysis

GraphPad Prism (v.5, GraphPad Software) was used for graph illustrations and statistical analysis. The level of significance was evaluated by one-way analysis of variance (ANOVA), using Dunnett’s multiple comparison test between non-treated samples and treated samples. The statistical significance level was set to a P value of ≤ 0.01.

## Results

### DnaA-PNA inhibits cell growth in an oriC-dependent manner, through the inhibition of DnaA translation

**Figure 1:**
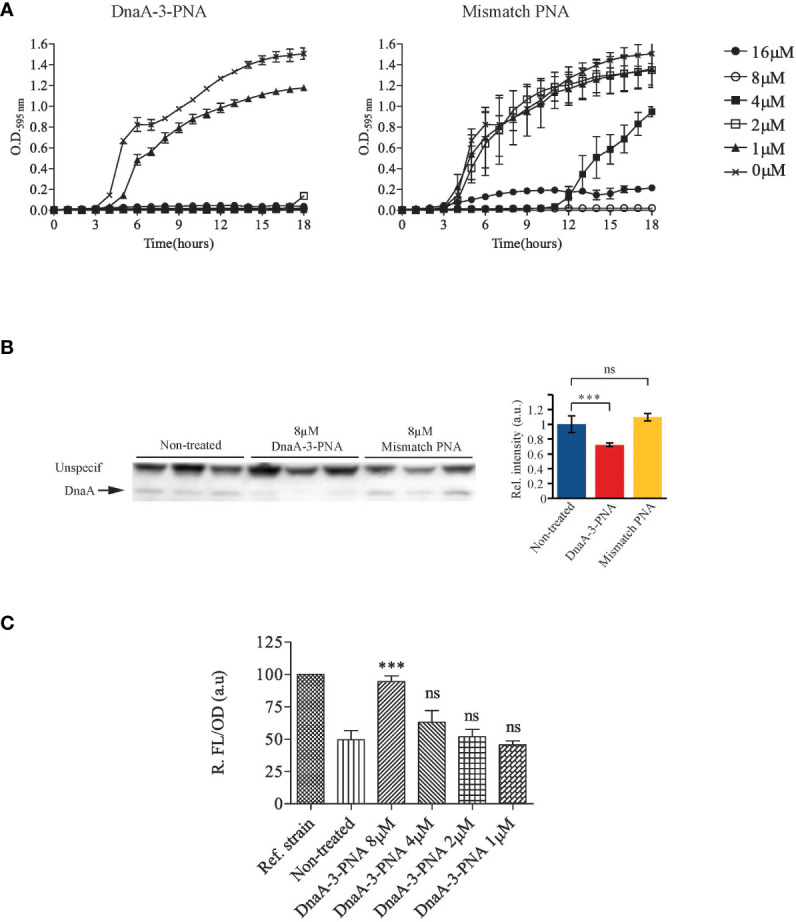
DnaA-3-PNA inhibits cell growth, through the inhibition of DnaA translation. **(A)** Anti-DnaA PNA-BPPs inhibits bacterial growth. Overnight cultures of wild-type cells were diluted to 10^5^ cfu/mL in MHB-I and then appropriate concentrations of the indicated PNA-BPPs were added. Cells were incubated at 37°C for 18 h, while turbidity was measured at OD_595_. **(B)** Anti-DnaA PNA-BPPs inhibits *dnaA* translation. Cell extracts for protein immunoblotting was collected from cells growing exponentially in AB minimal medium supplemented with glucose and casamino acids, treated with 8 μM of either DnaA-3-PNA or 2x-mismatch KFF. Cell extracts were collected at OD_450_ = 0.4-0.5 and proteins were separated in SDS-PAGE gels. DnaA protein was detected by immunoblotting. The nonspecific band seen with the DnaA antibody was used as loading control. Relative band intensity relative to wild type is shown as a bar graph representing the mean ± SD based on the band intensity; ns, not significant; ***, P<0.01. **(C)** DnaA-3-PNA specificity confirmed in an *oriC*/DnaA specific inhibitor screen. Overnight cultures of ALO5429 were diluted to 10^5^ cfu/mL in AB minimal medium supplemented with glucose and casamino acids and then appropriate concentrations of DnaA-3-PNA were added. Cells were incubated at 37°C for 18 h. The cells were washed in 10% NaCl and then turbidity and fluorescence were measured. The reference strain ALO5125 does not contain the *cI* carrying mini-chromosome pRNK6 and hence, represent the maximal fluorescence obtainable in this system.

To target *dnaA* mRNA translation, three anti-DnaA-PNAs were designed complementary to a 10-nucleotide region of the *dnaA* mRNA transcript, proximal to the ribosomal binding site and the GUG start codon (DnaA-1-, DnaA-2-, and DnaA-3-PNA). Each PNA was conjugated to the well-characterized lysine-rich (KFF)_3_K BPP (**Table 1**). Note that this carrier peptide is degraded upon entry into the bacterial cell so that only PNA or KFF-PNA enters the bacterial cell (Yavari et al., 2021). Therefore, the BPP moiety is not considered a hindrance in the hybridization between PNA and its mRNA target. Antimicrobial activity was determined in wild-type *E. coli* cells (MG1655), and all anti-DnaA PNA-BPPs inhibited cell growth at minimal inhibitory concentrations (MICs) of 2–4 μM (**Table 1**; **Figure 1A**; **Supplementary Figure S2A**). We decided to proceed with the DnaA-3-PNA which had a MIC of 2 μM, and a mismatch PNA control was designed based on its sequence, but with two bases interchanged (mismatch KFF). The mismatch KFF had a MIC of 16 μM (**Table 1**; **Figure 1A**) which is consistent with previous observations made from scrambled/mismatched PNA sequences of a similar length, indicating a degree of toxicity of PNA and/or the carrier peptide itself (Campion et al., 2021; Popella et al., 2022).

Western blot analysis revealed that DnaA protein levels were reduced by about 30% by DnaA-3-PNA treatment (**Figure 1B**; **Supplementary Figure S2B**) while the corresponding mismatch PNA did not significantly affect DnaA protein levels. The modest decrease in DnaA level should be seen in light of the stability of the protein with a half-life of >24 h (Torheim et al., 2000). Because samples for Western blots were taken at a much higher cell density (OD450 = 0.01) than those used for MIC determinations (OD600 = 0.0002), 8 μM of DnaA-3-PNA was used. The two other anti-DnaA-PNA, DnaA-1-PNA and DnaA-2-PNA, reduced the level of DnaA protein to about the same level as DnaA-3-PNA (**Supplementary Figure S2B**), strongly supporting the conclusion that anti-DnaA PNA-BPP specifically inhibits translation of the *dnaA* mRNA.

To show that growth inhibition was through the prevention of DnaA-mediated chromosome replication initiation at *oriC*, we tested anti-DnaA PNA-BPPs in a recently developed DnaA-inhibitor screen (Klitgaard and Løbner-Olesen, 2019). This assay utilizes *ΔrnhA, ΔoriC E. coli* cells, which initiate chromosome replication from *oriK* sites independent of DnaA and *oriC* (de Massy et al., 1984; Maduike et al., 2014), in a process called *constitutive stable DNA replication* (cSDR) (Kogoma, 1997). The screening strain, ALO5429, contains a chromosomally encoded GFPmut2 under lambda P_R_-promoter control and an *oriC-* and DnaA-dependent mini-chromosome (pRNK6) carrying a constitutively expressed lambda phage *cI* repressor gene. In this screen, any compound that inhibits the DnaA function or *oriC* strand opening, will lead to loss of the mini-chromosome and hence, the lambda CI repressor, which in turn results in an increase in fluorescence, without compromising cell viability. Cells treated with DnaA-3-PNA exhibited a concentration-dependent increase in relative fluorescence, indicating that replication of the mini-chromosome was inhibited (**Figure 1C**). Again, the two other anti-DnaA-PNA, DnaA-1-PNA and DnaA-2-PNA, behaved as DnaA-3-PNA (**Supplementary Figure S2C**). The degree of inhibition was similar to that observed by the expression of a cyclic DnaA domain I-derived peptide known to inhibit DnaA function (Kjelstrup et al., 2013). In summary, these data are consistent with the conclusion that DnaA-3-PNA specifically targets DnaA protein expression and inhibits bacterial growth in an *oriC-*dependent manner. Note that bacterial sensitivity to a reduction in the synthesis of different essential proteins varies, and therefore, MIC values are not necessarily proportional to their reduction level (Popella et al., 2022).

### Anti-DnaA PNA-peptides prevent the initiation of chromosome replication

**Figure 2:**
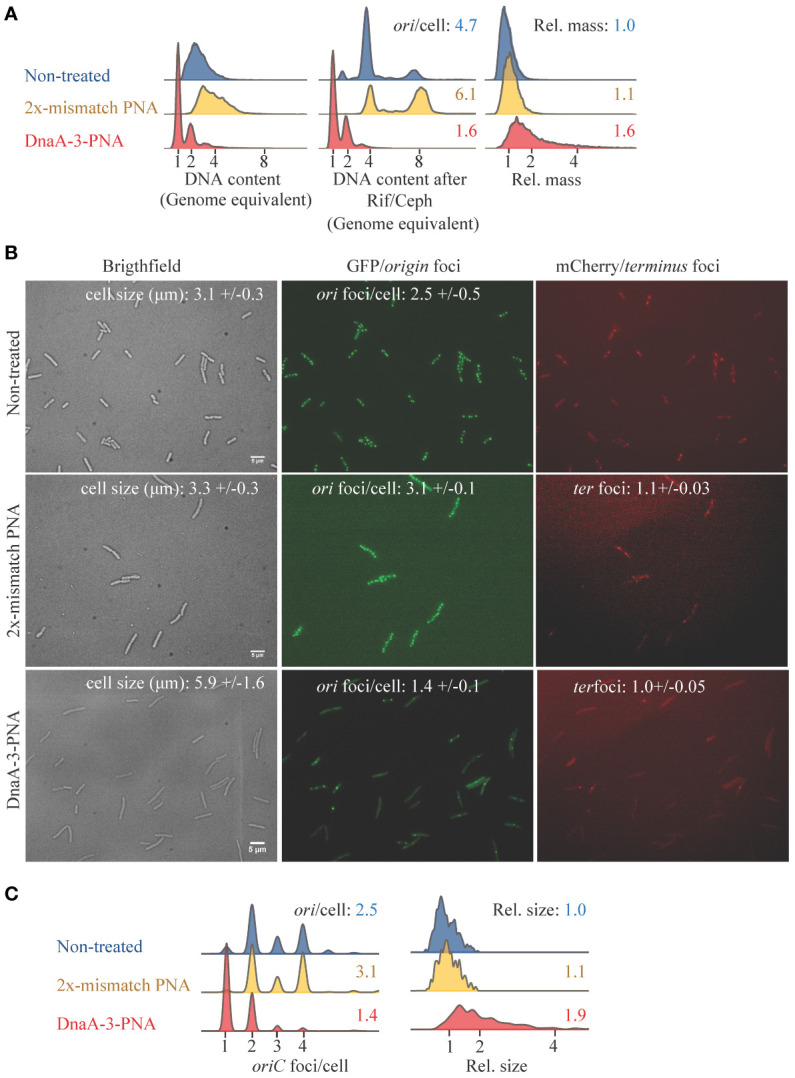
DnaA-3-PNA inhibits initiation of chromosome replication. **(A)** Cell-cycle analysis of cells treated with anti-DnaA PNA-BPPs. MG1655 were grown exponentially in AB minimal medium supplemented with glucose and casamino acids, diluted to OD_450_ = 0.005, and exposed to DnaA-3-PNA or mismatch KFF (10 μM) for 2 h before sample collection for flow cytometry. The following are shown: cell size distributions (a.u.) of exponentially grown cells, DNA content of cells, and DNA content of cells treated with rifampicin and cephalexin for chromosome replication to be completed. Each panel represents 30–50,000 cells. **(B)** Cell morphology and localization of *ori* and *ter* regions of DnaA-3-PNA and 2x-mismatch KFF-treated cells. Strain ALO4223 was grown exponentially in AB minimal medium supplemented with glucose and casamino acids, diluted to OD_450_ = 0.005, and exposed to DnaA-3-PNA or 2x-mismatch KFF (10 μM) or left untreated for 2 (h) Scale bar is 5 μm. Cell size and cell cycle parameters given as *ori* foci/cell and *ter* foci were inserted. These values were based on pooled counts of 2–3 independent determinations: Non-treated (n= 256, 211, and 128); DnaA-3-PNA (n= 222, 211, and 151); mismatch-PNA (n= 168 and 117). **(C)** *ori* foci/cell and relative cell size were determined for each growth/treatment condition and visualized as histograms.

Flow cytometry was used to assess the effect of DnaA-3-PNA on chromosome replication (Lobner-Olesen et al., 1989). During growth in minimal medium supplemented with glucose and casamino acids, untreated cells were uniform in mass and DNA content, with the majority containing four chromosome equivalents, after being treated with rifampicin and cephalexin to block replication initiation and cell division, respectively (**Figure 2A**). These chromosome equivalents represent the number of chromosomal origins (*oriC*) in each cell, at the time of drug treatment (Boye and Lobner-Olesen, 1991). The average number of *origins*/cell was 4.7 (**Figure 2A**). Cells treated for 2 h with DnaA-3-PNA had a lower DNA content, with the majority of cells containing one fully replicated chromosome and a few cells with two fully replicated chromosomes.

The appearance of fully replicated chromosomes in the absence of rifampicin/cephalexin treatment is usually observed in cells entering stationary phase and ceasing chromosome replication (Boye and Lobner-Olesen, 1991). Because the optical density of the DnaA-3-PNA treated cultures never exceeded OD_450 _= 0.2, present data suggest that cells never entered stationary phase but that treatment with DnaA-3-PNA led to cessation of chromosome replication initiation and continued cell division, eventually generating cells containing one fully replicated chromosome (**Figure 2A**). The increase in cell size upon 2-h treatment with DnaA-3-PNA also suggests that once formed, the 1-chromosome cells continue to increase in size without further initiations (**Figure 2A**). A similar pattern was observed for *dnaA46* cells shifted to non-permissive temperature (see below). Consequently, treatment with rifampicin and cephalexin did not alter the DNA distribution of DnaA-3-PNA-treated cells (**Figure 2A**). The average cell size increased when cells were treated with DnaA-3-PNA for 2 h (**Figure 2**) and continued to increase upon longer periods of treatment (**Supplementary Figure S3**). The decrease in average *origins*/cell and increase in cell size led to an overall decrease in origin concentration (*origins*/cell mass) for cells treated with DnaA-3-PNA. Cells treated with the mismatch PNA resembled untreated cells. Collectively, these observations strongly suggest that cells treated with DnaA-3-PNA fail to initiate new rounds of chromosome replication, which results in the appearance of fully replicated chromosomes and a decrease in the average number of *origins*/cell. This leads to a delay in cell division and an increase in relative cell mass.

We proceeded to directly visualize the effect of DnaA-3-PNA in an *ori* and *ter* tagged strain, as previously described (Charbon et al., 2014). The non-treated cells were uniform in appearance, and the majority of cells observed contained two or four origin foci per cell with a mean of 2.5 (**Figures 2B, C**). Cells treated with DnaA-3-PNA for 2 h became heterogeneous in size, with the majority of cells being enlarged. The majority of cells contained one *origin* foci/cell only (**Figures 2B, C**). The average number of *ter* foci per cell remained close to one irrespective of DnaA-3-PNA treatment (**Figure 2B**). Cells treated with mismatch PNA were very similar to non-treated cells (**Figures 2B, C**).

To allow comparison of DnaA-3-PNA-treated wild-type cells to cells limited in activity or amount of the DnaA protein, we used a thermo-sensitive *dnaA* mutant (DnaA46) and a DnaA-depletion assay, respectively.

**Figure 3:**
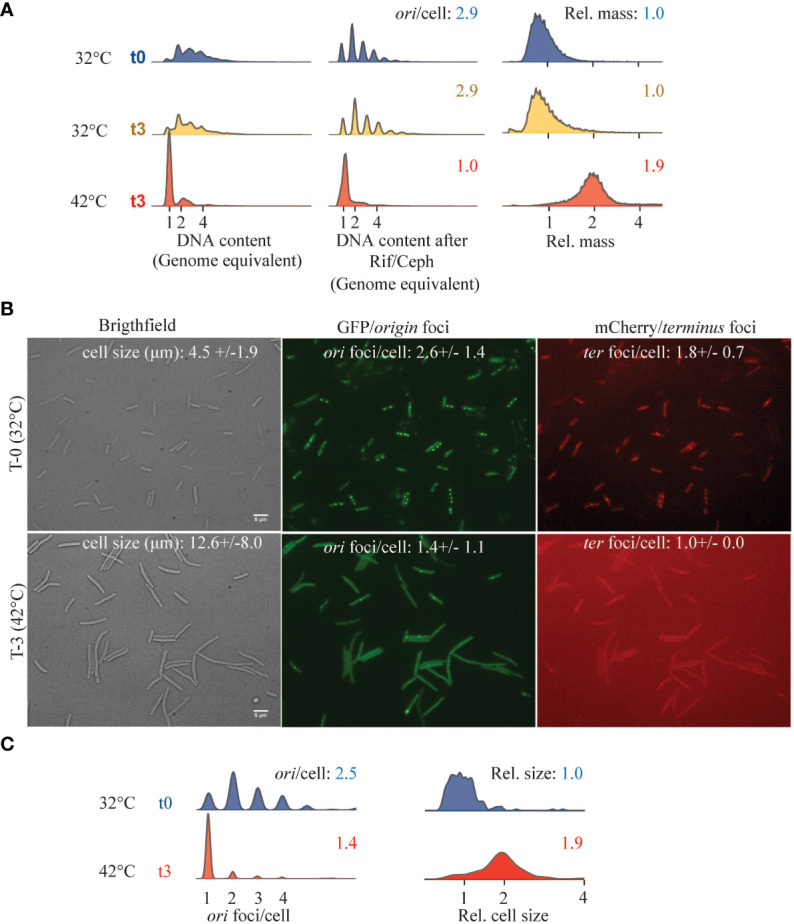
Cell cycle parameters of DnaA46 mutants at non-permissive temperature. ALO8290 cells were grown exponentially in AB minimal medium supplemented with glucose and casamino acids, diluted to OD_450_ = 0.05 (T = 0), and then split with one half remaining at 32°C and the other half was incubated at 42°C. At the indicated time points, samples were collected for flow cytometry and fluorescence microscopy. **(A)** Histograms of cell size distribution (a.u.), DNA content, and DNA content of cells treated with rifampicin and cephalexin. Each panel represents 30–80,000 cell events. **(B)** *In vivo* visualization of *ori* and *ter* foci, alongside phase contrast images at 32°C (T = 0) and following 3 h at 42°C (T = 3). Cell size and cell cycle parameters given as *ori* foci/cell and *ter* foci/cell were inserted. Cell length and foci/cell were determined based on n = 197–545 counted cells of each condition, individually (32°C T0; n = 234) and (42°C; n = 307). Scale bar is 5 μm. **(C)** Relative cell size and *ori* foci/cell were determined for each growth condition indicated and visualized as histograms.

The temperature-sensitive DnaA46 protein contains A184V and H252Y substitutions, both in the domain responsible for ATP binding (Carr and Kaguni, 1996). Consequently, the DnaA46 protein binds neither ATP nor ADP at any temperature, yet the DnaA protein is functional and is capable of initiating DNA replication from *oriC* at permissive temperature, albeit with a loss of coordination of initiations (Skarstad et al., 1986, 1988). When DnaA46 cells were shifted to non-permissive temperature, cells ceased to initiate new rounds of chromosome replication, which resulted in the appearance of fully replicated chromosomes and a decrease in the average number of *origins*/cell (**Figure 3A**). As a consequence of the cessation of initiation of DNA replication, cell division was delayed, leading to an increase in relative cell mass (**Figure 3A**). Similarly, when visualized *in vivo*, cells shifted to non-permissive temperature became elongated and contained mainly one *origin* foci/cell (**Figures 3B, C**).

**Figure 4:**
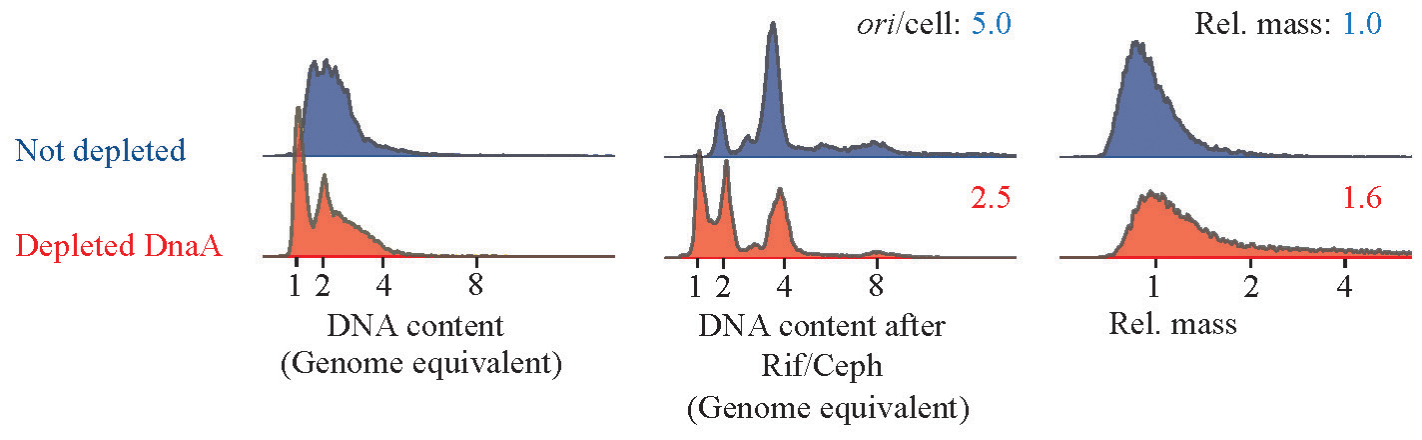
Cell-cycle profile of DnaA depleted cells. Strain ALO8528 was grown exponentially in AB minimal medium supplemented with glycerol, casamino acids, and tetracycline. At T = 0, the culture was split, one portion with 1-mM IPTG added to initiate DnaA depletion and the other portion was left untreated. Cells were propagated for approximately 12 mass doublings in the same medium while diluting cells whenever OD_450_ exceeded 0.2. Finally, samples were collected at OD_450_ = 0.2 for flow cytometry analysis. Each panel represents 30–80.000 cells.

To deplete cells for DnaA, we used *ΔdnaA* mutant cells carrying the *dnaA* gene expressed from its native promoter on a low copy number R1-based plasmid. The cells also carried the pSC101-derived plasmid pKG339, which, upon IPTG induction, expressed the antisense RNA *CopA* that inhibits the R1 plasmid replication (Jensen et al., 1995). Upon IPTG addition, the replication of the R1-based plasmid carrying *dnaA* ceases while cell growth and division continue (Jensen et al., 1995). Over time, the plasmid carrying *dnaA* is lost, leading to dilution of the DnaA protein. We observed that IPTG induction resulted in the appearance of fully replicated chromosomes (**Figure 4**), a phenotype similar to that of DnaA46 cells at non-permissive temperature and to DnaA-3-PNA-treated cells. However, further treatment with rifampicin and cephalexin revealed that replication cessation by depletion was somewhat partial (**Figure 4**). DnaA depletion also resulted in an increased cell size and hence, a reduced average number of *origins*/cell. These findings further support that treatment with anti-DnaA PNA does indeed result in DnaA depletion leading to growth inhibition.

## Discussion

### DnaA as an antimicrobial drug target

As a conserved and essential gene, DnaA has long been considered an excellent antimicrobial drug target (Grimwade and Leonard, 2019). The DnaA protein itself can be targeted at multiple levels such as DnaA ATPase activity, oligomerization, loading of the helicase or binding to *oriC*, and several *in vitro* screens have been developed to identify molecules that affect these activities (Sasaki et al., 1994; Johnsen et al., 2010; Charbon et al., 2018a; Klitgaard and Løbner-Olesen, 2019). An *in vitro* non-competitive inhibitor of ATP binding (3-Acetoxy-2.2’-bi-1H-indol) was discovered in 1994 (Sasaki et al., 1994), showing a IC_50_ of 0.04-mM in a DnaA-ATP binding assay. A derivative of this compound (3-[*N*-(11-carboxyundecyl)] carbamoylmethoxy-2.2´-bi-1H-indol) was later found to be more potent, with an IC_50_ value of 7 μM (Mizushima et al., 1996). However, the antibacterial activity and specificity of these drugs have never been reported. The expression of a cyclic DnaA domain I-derived peptide known to specifically inhibit DnaA oligomerization has been shown to arrest cellular growth (Kjelstrup et al., 2013). However, due to poor cell penetration of peptides, it remains to be seen whether the cyclic peptide could be used as an actual drug when provided extracellularly. Other attempts have been made using cell-based screens to find drugs that work in cell culture (Fossum et al., 2008; Johnsen et al., 2010; Charbon et al., 2018a; Klitgaard and Løbner-Olesen, 2019). However, none of the molecules identified were found to affect DnaA, indicating that the protein may be difficult to target. It is likely that drugs that affect DnaA activity would mimic ATP and in principle, target other ATP-binding proteins as well. Moreover, small molecules affecting protein–protein interaction are expected to be difficult to find (Grimwade and Leonard, 2019). The apparent ease of selection of intra or extragenic suppressor mutations further complicates the screening process (Charbon et al., 2018b). Targeting the expression of DnaA can circumvent these hurdles because the loss of/reduction in DnaA protein level results in the cessation of chromosome replication and inhibition of cell growth as observed here. Note that the effect of DnaA translation knockdown is not limited to DNA replication as other essential cellular processes may be affected as well (Menikpurage et al., 2021) since DnaA is also a global transcription factor. DnaA has been linked to the regulation of several genes encoding enzymes pivotal for essential processes such as deoxyribonucleotide synthesis through. *nrdAB* (Augustin et al., 1994; Gon et al., 2006; Olliver et al., 2010; Babu et al., 2017) and *guaB* (Tesfa-Selase and Drabble, 1992), DNA repair, through *uvrB* (Wurihan et al., 2018) and *polA* (Quinones et al., 1997), as well as playing a role in stress survival (Sass et al., 2022).

The present antisense compounds inhibited MG1655 growth at approximately 2 μM and demonstrated that *dnaA* translation is a functional antisense PNA target. However, on the road toward new antibiotics, a systematic structure activity approach identifying the most sensitive PNA sequence target sites in the *dnaA* mRNA as well as the most effective BPP must be carried out in order to optimize potency, specificity, cytotoxicity, and biostability, and eventually *in vivo* properties. Indeed, a large variety of arginine/guanidinium BPPs that also allow *in vivo* efficacy studies have already been identified (Goltermann et al., 2019; Frimodt-Moller et al., 2021; Iubatti et al., 2022).

An antimicrobial effect has previously been observed for the control PNA-peptides with scrambled/mismatch sequences, which have been in part attributed to the activation of envelope stress response pathways (Popella et al., 2022).

## Conclusion

Lysine-rich (KFF)_3_K PNA-BPPs designed to target the initiation of DNA replication, by inhibiting the expression of the initiator protein DnaA, exhibited micromolar antibacterial activity toward MG1655, and the treated cells had decreased DnaA protein levels that led to growth inhibition dependent on *oriC*. Furthermore, cells treated with DnaA-3-PNA exhibited hallmarks associated with chromosome replication inhibition similar to that observed when DnaA activity was compromised (*i.e.* in DnaA46 cells at non-permissive temperature), or when DnaA was depleted. Collectively, the data provide strong genetic and phenotypic evidence for the inhibition of initiation of chromosome replication in cells where *dnaA* translation was inhibited by specific PNA-BPP, and thus, identifies *dnaA* as a *bona fide* antibacterial target using antisense-PNA technology.

## Data availability statement

The original contributions presented in the study are included in the article/**Supplementary Material**. Further inquiries can be directed to the corresponding author.

## Author contributions

CC: Conceptualization, Writing – original draft, Writing – review & editing, Formal analysis, Investigation, Methodology. GC: Conceptualization, Investigation, Methodology, Writing – original draft, Writing – review & editing. PN: Conceptualization, Funding acquisition, Investigation, Methodology, Project administration, Resources, Writing – original draft, Writing – review & editing. AL: Conceptualization, Project administration, Resources, Supervision, Writing – original draft, Writing – review & editing.
