# Supplementary material for: Targeting synthesis of the Chromosome Replication Initiator Protein DnaA by antisense PNA-peptide conjugates in Escherichia coli
Source: Front Antibiot. 2024 Apr 8;3:1384390. doi: 10.3389/frabi.2024.1384390 (PMC11732032; doi:10.3389/frabi.2024.1384390)
Supplement: Supplementary file 1 [file DataSheet_1.pdf]

Supplementary Material

Targeting synthesis of the Chromosome Replication Initiator Protein DnaA by antisense PNA-peptide conjugates in *Escherichia coli*

Christopher Campion[1,2], Godefroid Charbon[2], Peter E. Nielsen[1] and Anders Løbner-Olesen[2*]

[1]Department of Cellular and Molecular Medicine, University of Copenhagen, 2200 Copenhagen N, Denmark.

[2]Department of Biology, University of Copenhagen, 2200 Copenhagen N, Denmark.

*** Correspondence:**
Anders Løbner-Olesen
Lobner@bio.ku.dk

Supplementary Figures and Tables

Table S1. Bacterial strains and plasmids.

Strain/Vectors	Genotype	Reference/source
MG1655	F^{-}λ-*rph-1*.	(Guyer, Reed et al. 1981)
ALO4223	inter(*ydeU;ydeK*)::*parS*pMT1, inter(*ilvA;ilvY*)::*parS* P1::Kan, *attTn7*::*pTrc*- mCherry-pMT*parB* - GFP-P1*parB*[a]	(Charbon, Bjorn et al. 2014)
ALO8290	*dnaA46*[b]	This work
ALO5429	*ΔoriC* (Cam^{R}) *ΔrnhA*, *attB*::PR-GFP*mut2* (Kan^{R}), pRNK6($Strep^{R}$)[a] Replication inhibition strain.	(Klitgaard and Løbner-Olesen 2019)
ALO5125	*ΔoriC* (Cam^{R}) *ΔrnhA*, *attB*::PR-GFP*mut2* (Kan^{R})[a] Replication inhibition reference strain.	(Klitgaard and Løbner-Olesen 2019)
ALO2342	*dnaA46, tnaA:Tn10*[a]	This work
ALO8528	*ΔdnaA*::cat / pJEL-*dnaA* and pKG339[a]	This work
TC3874	*araD139*, *del(ara-leu)7679, del(lac)X74,* *dnaA: :cat, galK, galU, hsdR, rnhA-373, rpsl, thi*	(Ingmer and Atlung 1992)

[a] Genotype otherwise as MG1655

[b] Genotype otherwise as ALO4223

Table S2. PNA off-targets

DnaA-1-PNA		DnaA-2-PNA		DnaA-3-PNA		Mismatch-PNA	
Gene	Location	Gene	Location	Gene	Location	Gene	Location
dnaA	+7	*dnaA*	+6	*purA*	CDS	*dppA*	CDS
efeB	CDS			*dnaA*	+5	*gltB*	CDS
				ybfB	CDS	*yggF*	CDS
						yedN	CDS-pseudogene
						ydjG	CDS
						ybeU	CDS
						citE	CDS
						yajR	ca. 40bp upstream

All PNA sequences were subject to BLAST using the NCBI BLASTn tool (https://blast.ncbi.nlm.nih.gov/Blast.cgi). Genomic sites with 100% homology match are listed, "+/-" refers to upstream/downstream of the first base pair of the closest reading frame, "CDS" refers to homology sites within a coding sequence.

A.

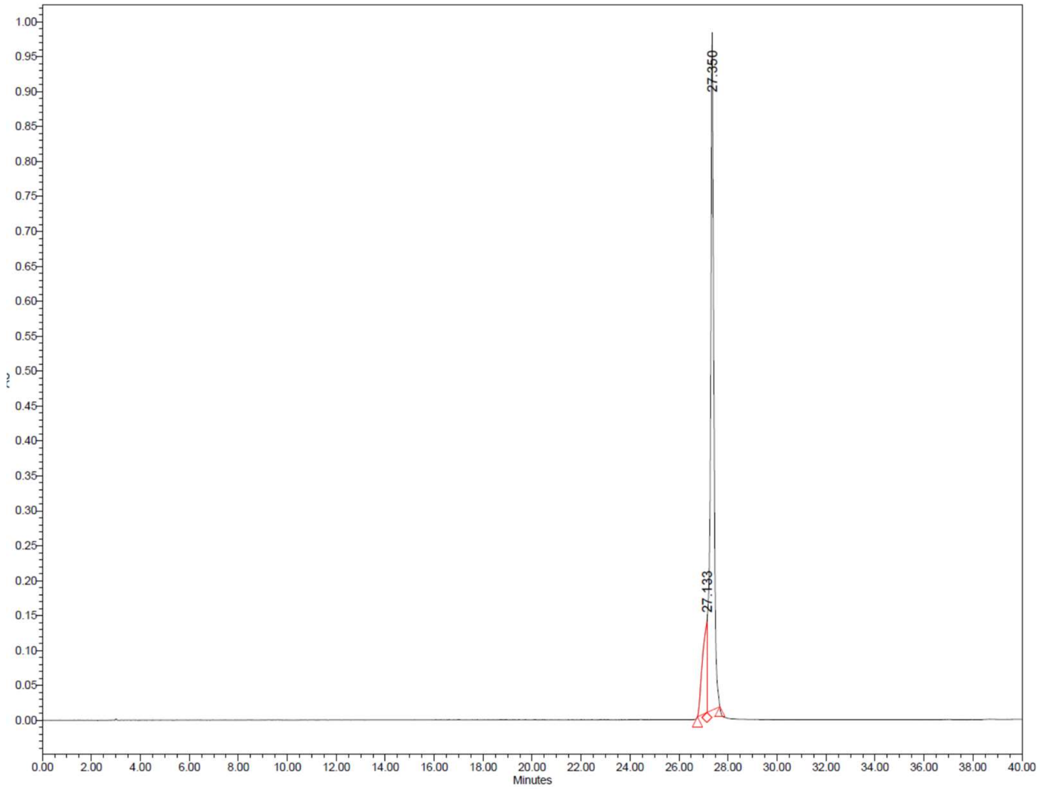
27.350
27.133
Minutes

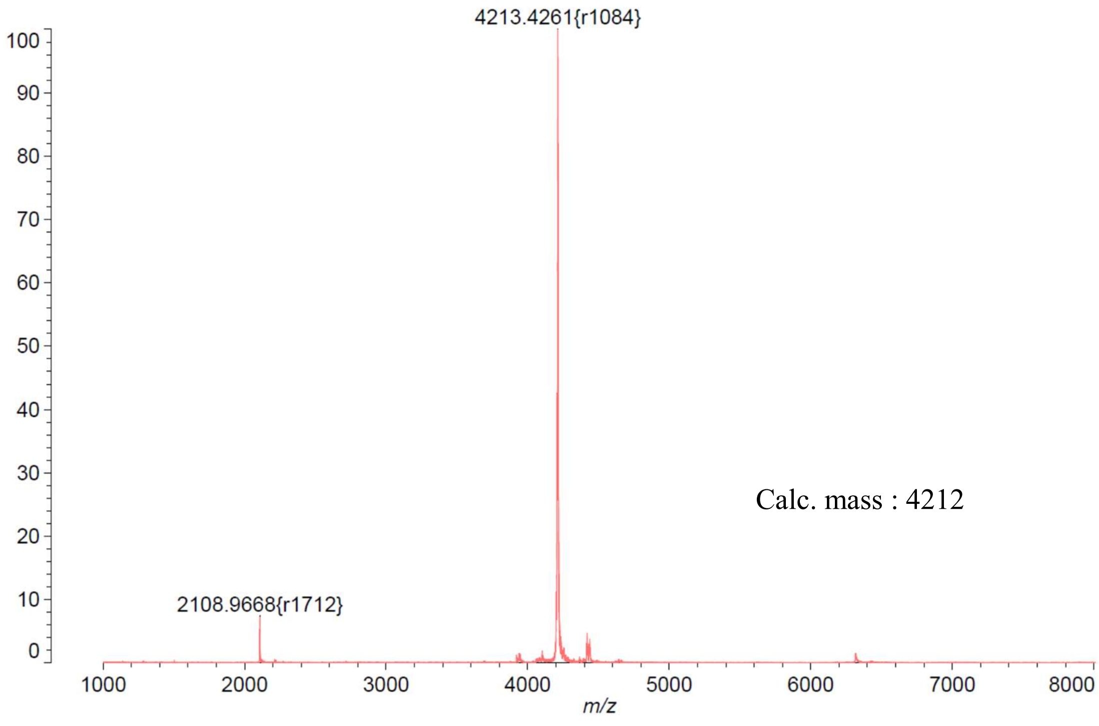
4213.4261{r1084}
2108.9668{r1712}
Calc. mass : 4212
m/z

B.

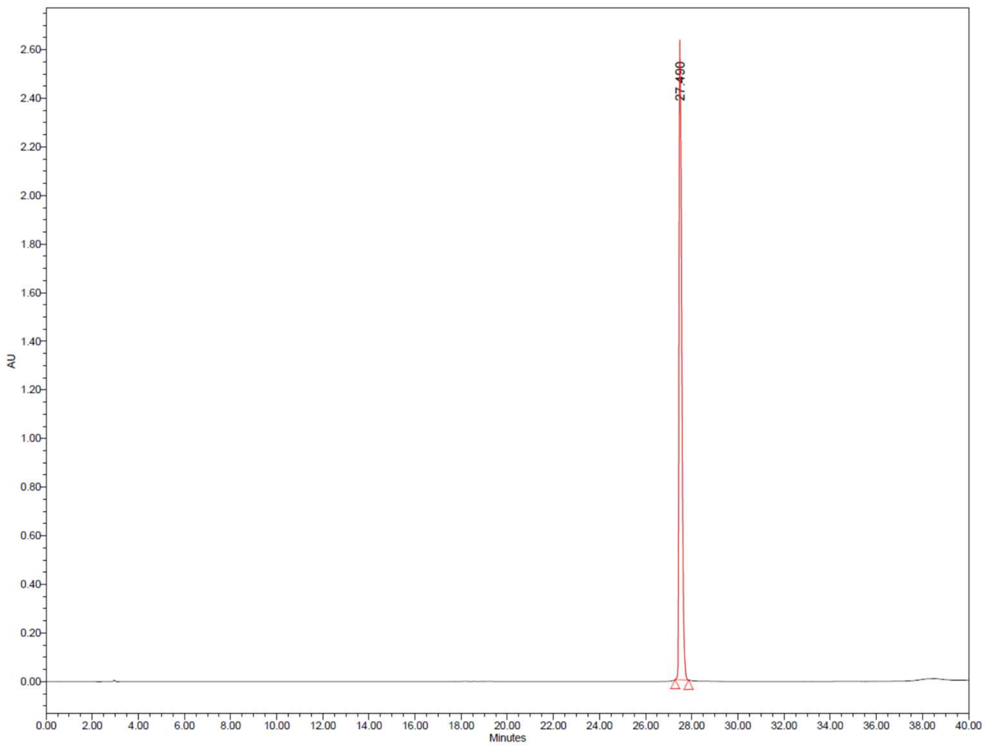

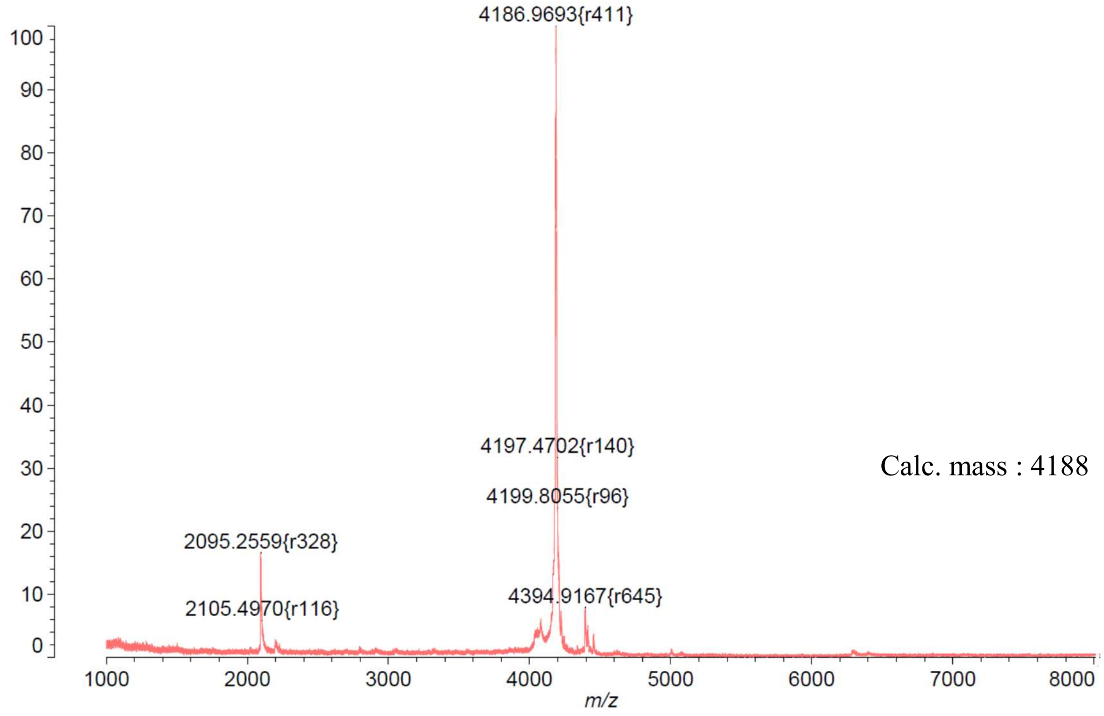

Calc. mass : 4188

C.

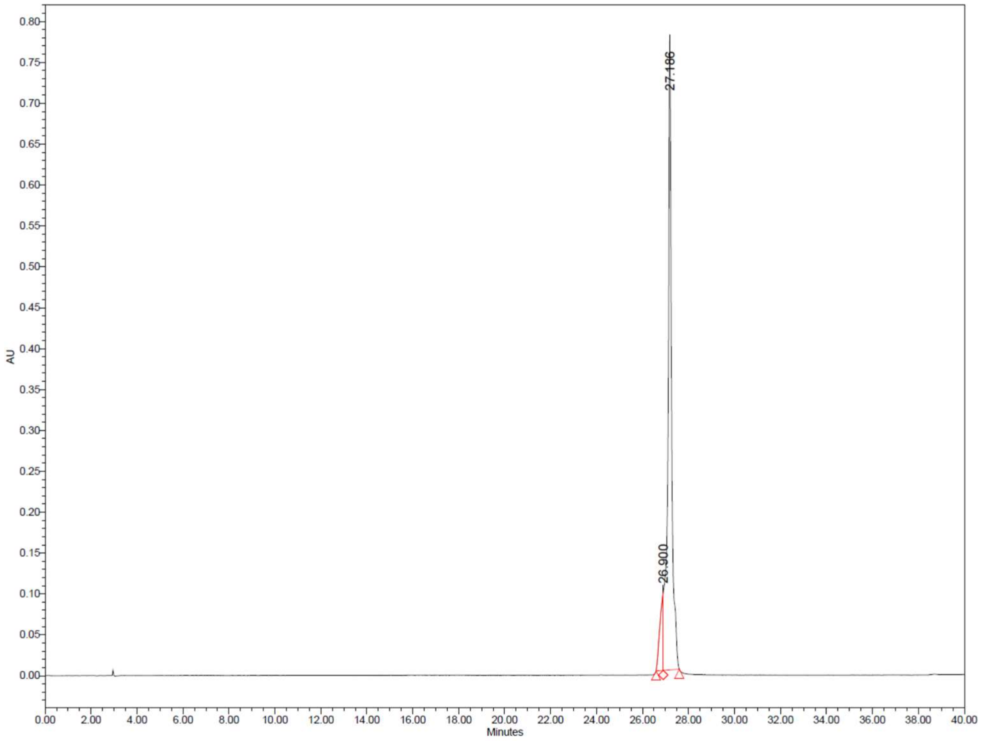

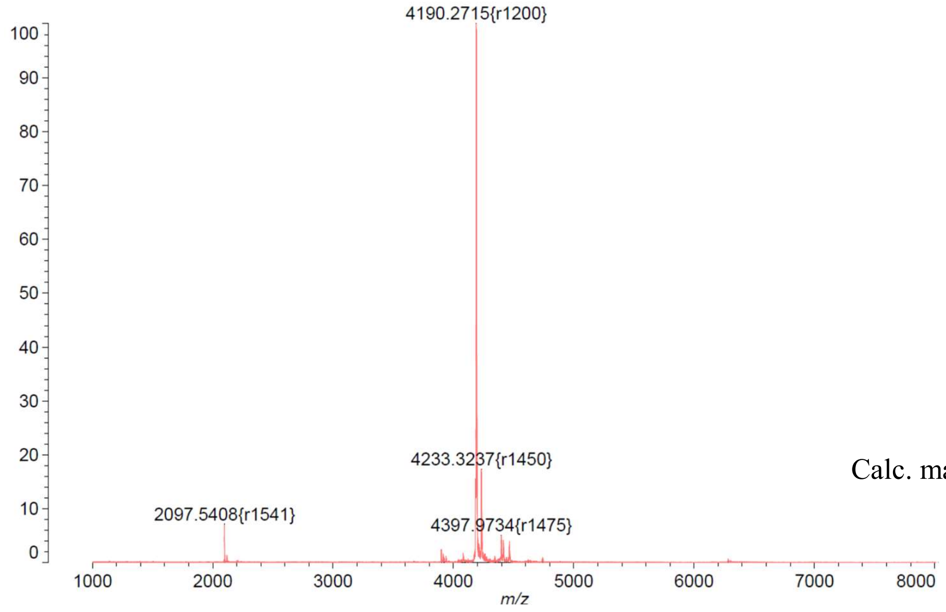

Calc. mass : 4188

D.

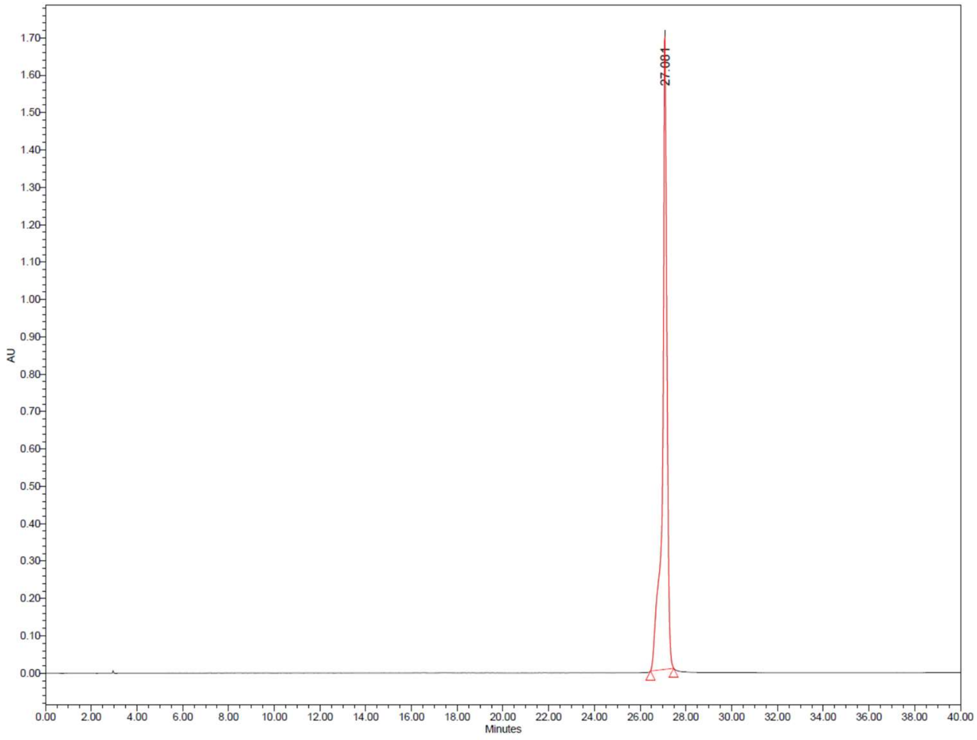

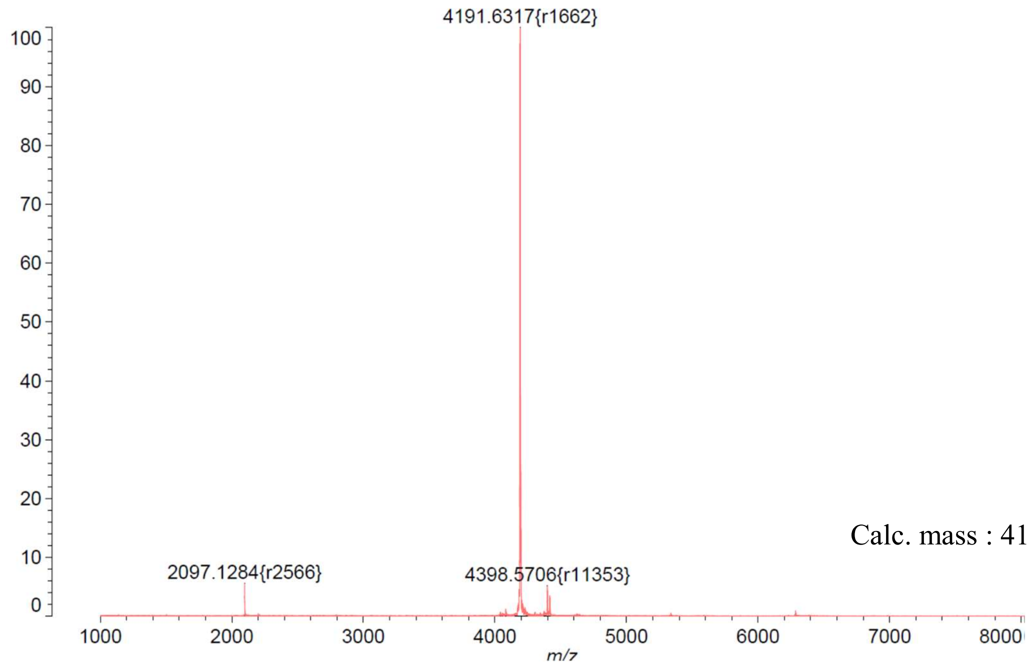

Calc. mass : 4188

Supplementary Figure 1. Purity of the PNA-peptide conjugates used

A. HPLC and MALDI-TOF MS of DnaA-1-PNA
B. HPLC and MALDI-TOF MS of DnaA-2-PNA
C. HPLC and MALDI-TOF MS of DnaA-3-PNA
D. HPLC and MALDI-TOF MS of mismatch-PNA

HPLC, high performance liquid chromatography; MALDI-TOF, matrix assisted laser desorption ionization-time of flight.

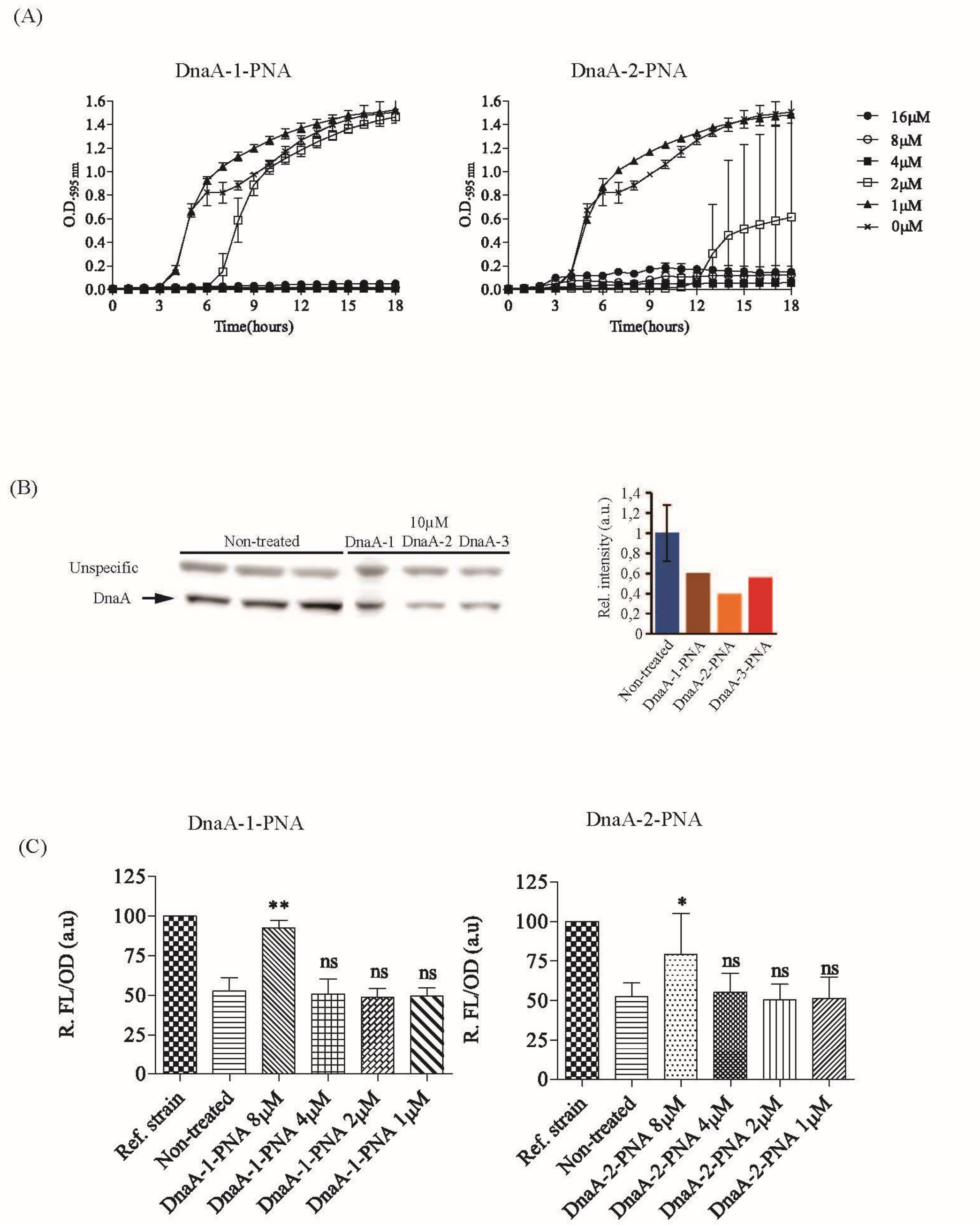

Supplementary Figure 2. DnaA-1 KFF and DnaA-2 KFF inhibits cell growth in a DnaA and *ori* dependent manner.

A) Anti-DnaA PNA-BPPs inhibits bacterial growth. Overnight cultures of MG1655 were diluted to 10^5 cfu/ml in MHB-I and added appropriate concentrations of the indicated PNA-BPPs. Cells were incubated at 37°C for 18 hours, while turbidity was measured at OD_{595}.

B) Anti-DnaA PNA-BPPs inhibits *dnaA* translation. Cell extracts for protein immunoblotting was collected from cells growing exponentially in AB minimal medium supplemented with glucose and casamino acids, treated with 10 μM of the indicated PNA-BPPs. Cell extracts were collected at OD_{450} = 0.4-0.5 and proteins separated in SDS-PAGE gels. DnaA protein wasdetected by immunoblotting. Relative band intensity relative to wild type is shown as a bar graph representing the mean±SD based on the band intensity, ns, not significant; ***, P<0.01.

C) DnaA-1-PNA and DnaA-2-PNA specificity confirmed in an *oriC*/DnaA specific inhibitor screen. Overnight cultures of ALO5429 were diluted to 10^5 cfu/ml in AB minimal medium supplemented with glycerol and casamino acids and added appropriate concentrations of DnaA-3-PNA. Cells were incubated at 37°C for 18 hours. The cells were washed in 10 % NaCl and turbidity and fluorescence measured. The reference strain ALO5125 does not contain the *cI* carrying minicromosome pRNK6 and hence represent the maximal fluorescence obtainable in this system.

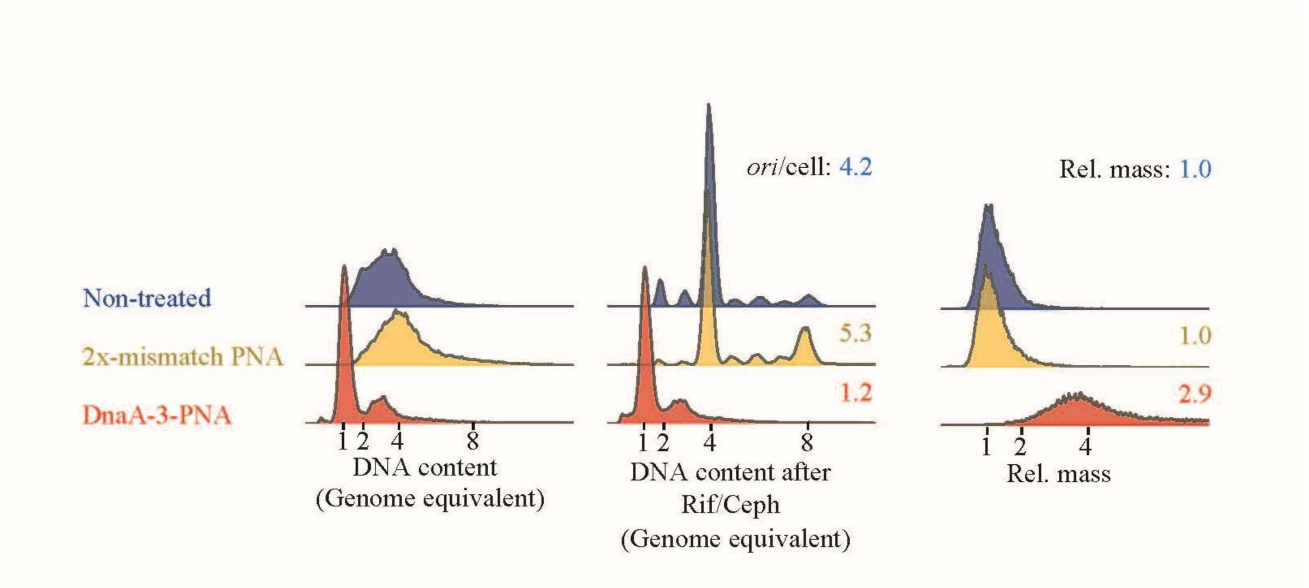

Supplementary Figure 3. DnaA-3-PNA inhibits initiation of chromosome replication.

MG1655 were grown exponentially in in AB minimal medium supplemented with glucose and casamino acids, diluted to OD_{450} = 0.005 and exposed to DnaA-3-PNA or corresponding mismatch PNA (10 µM) for four hours before sample collection for flow cytometry. Shown are cell size distributions (a.u.) of exponentially grown cells, DNA content of cells and DNA content of cells treated with rifampicin and cephalexin for chromosome replication to complete. Each panel represents 30-50.000 cells.
